# Distinct Patterns of Automatic and Controlled Incongruent Information Processing in the Human Brain

**DOI:** 10.3389/fnhum.2022.836374

**Published:** 2022-05-06

**Authors:** Jialin Du, Yu Zhu, Chengtian Zhao, Dongju Yang, Tao Yu, Xiaohua Zhang, Liankun Ren, Yuping Wang

**Affiliations:** ^1^Department of Neurology, Xuanwu Hospital, Capital Medical University, Beijing, China; ^2^Department of Functional Neurosurgery, Beijing Institute of Functional Neurosurgery, Xuanwu Hospital, Capital Medical University, Beijing, China; ^3^Institute of Sleep and Consciousness Disorders, Beijing Institute for Brain Disorders, Beijing, China; ^4^Beijing Key Laboratory of Neuromodulation, Beijing, China

**Keywords:** incongruent information processing, stereoelectroencephalography, event-related potential, frontoparietal control network, default mode network

## Abstract

It is a fundamental ability to discriminate incongruent information in daily activity. However, the underlying neural dynamics are still unclear. Using stereoelectroencephalography (SEEG), in this study, we investigated the fine-grained and different states of incongruent information processing in patients with refractory epilepsy who underwent intracranial electrode implantation. All patients performed a delayed match-to-sample paradigm in the sequential pairs of visual stimuli (S1 followed by S2). Participants were asked to discriminate whether the relevant feature of S2 was identical to S1 while ignoring the irrelevant feature. The spatiotemporal cortical responses evoked by different conditions were calculated and compared, respectively, in the context of brain intrinsic functional networks. In total, we obtained SEEG recordings from 241 contacts in gray matter. In the processing of irrelevant incongruent information, the activated brain areas included the superior parietal lobule, supramarginal gyrus, angular gyrus, inferior temporal gyrus, and fusiform gyrus. By comparing the relevant incongruent condition with the congruent condition, the activated brain areas included the middle frontal gyrus, superior temporal gyrus, middle temporal gyrus, posterior superior temporal sulcus, and posterior cingulate cortex. We demonstrated the dynamics of incongruent information processing with high spatiotemporal resolution and suggested that the process of automatic detection of irrelevant incongruent information requires the involvement of local regions and relatively few networks. Meanwhile, controlled discrimination of relevant incongruent information requires the participation of extensive regions and a wide range of nodes in the network. Furthermore, both the frontoparietal control network and default mode network were engaged in the incongruent information processing.

## Introduction

We detect, discriminate, and select incongruent information in daily life, representing the fundamental ability of cognitive control to guide thoughts and actions according to internal intentions (Breukelaar et al., 2017). It refers explicitly to ignoring goal-interfered information and emphasizing task-relevant stimulus information through the attentional biasing of perceptual processing (Egner and Hirsch, 2005). Over the past few decades, efforts have been made to probe the dynamics of incongruent information processing. Accumulative evidence derived from neuroimaging and electrophysiological studies has shown that incongruent information processing involves activation and interactions among distributed cortical areas (Spreng et al., 2010; Cocchi et al., 2013; Cole et al., 2014). Compared with other methods, electroencephalography (EEG) offers an optimized approach due to the high time resolution.

At present, a range of evoked potentials in relation to incongruent information have been reported under various conditions using scalp EEG recordings. In the oddball or no-go/go task paradigms, the N2 component that was first described in the 1970s showing greater negative amplitude on incongruent trials than on congruent trials at ~200–350 ms after stimulus presentation was classically recognized. There is a general consensus that N2 is the essential functionally related cognitive control index (Larson et al., 2014; Xiao et al., 2020), which is likely to reflect incongruent monitoring (anterior N2) and interference processing (posterior N2) (Tian et al., 2014). Moreover, Kotchoubey and Kramer reported that a more significant negative potential with peak latencies of 200–300 ms was elicited in a memory search task when the memory set consisted of only single-item trials, in which the comparison and probe stimulus occurred in immediate succession (Kramer et al., 1991; Kotchoubey et al., 1996). Using sequential matching tasks in which participants were required to decide whether a second stimulus was the same or different from an initial stimulus, our prior series of studies consistently demonstrated that such a subcomponent of N2 was enhanced when the second stimulus in a pair did not match the first one (Wang et al., 1998, 2000). The findings were further confirmed by Kimura et al. in irrelevant stimulus dimensions, showing that the irrelevant-change trials elicited a more prominent frontocentral N270 component than no-change trials (Kimura et al., 2006).

The identification of the N2 subcomponent is speculated to reflect the intrinsic template-matching process. A better understanding of the question of how the brain processes incongruent information is essential to investigate the course of cognitive control. In particular, the accurate location of neural sources at different stages is still to be refined. Direct recording of neural activity, which is available in the clinical presurgical evaluation of refractory epilepsy, provides a unique opportunity to elucidate the spatiotemporal processing on fine temporal (subsecond) and spatial (subcentimeter) scales. Considering that the intrinsic brain circuitry function is essential to implement the cognitive task, in this study, we addressed the relevant incongruent information processing in the context of the functional networks by means of stereoelectroencephalography (SEEG) using a delayed match-to-sample paradigm.

## Materials and Methods

### Participants and Implantation of SEEG Electrodes

In total, 10 right-handed patients (3 men and 7 women) with refractory epilepsy at Xuanwu Hospital Capital Medical University who underwent presurgical evaluation with SEEG implantation were included. All patients had normal intelligence (IQ score above 80) and standard or corrected-to-normal vision. The SEEG implantation plan was determined based on clinical grounds. SEEG electrodes (HuaKe HengSheng, Beijing, China) were stereotactically implanted under general anesthesia. Each SEEG electrode consisted of a cylinder, 0.8 mm in diameter, and contained 10–16 contacts of 2 mm in length separated by 1.5 mm. The implanted number and placement of the electrodes were based solely on medical considerations. The procedure has been described in our previous study (Ren et al., 2020). After surgery, long-term monitoring was performed for ~7–14 days to capture the habitual epileptic seizures.

The research protocol was approved by the Institutional Review Board Committee of Xuanwu Hospital Capital Medical University, in accordance with the ethical standards of the Declaration of Helsinki. Patients were thoroughly informed about the purposes of the study and gave written informed consent.

### Reconstruction of SEEG Electrodes

To determine the precise location of the SEEG electrodes, postsurgery CT scans were linearly and subsequentially non-linearly co-registered to the template T1-MRI image (MNI ICBM2009b NLIN, Asym) using Advanced Normalization Tools (ANTs, a toolbox that has been integrated into LEAD-DBS software, http://www.lead-dbs.org) (Horn et al., 2019). Then, the depth electrodes were readily detected three-dimensionally by visual inspection after normalization to template MNI, and contact locations were reconstructed using MATLAB (MathWorks Inc., Natick, MA, USA). This method has been validated in our previous study (Ren et al., 2020). All electrodes were automatically detected and classified according to parcellation of gray and white matter. Only contacts located in the gray matter were further analyzed.

Electrodes were categorized into the Yeo atlas 17-network (Yeo et al., 2011, 2014), according to the regions of interest as defined in the previous studies. In addition, the hippocampus was merged as the 18th cortical region.

### Experimental Procedure and SEEG Recording

After 3 days of electrode implantation surgery, the patient performed the task in a dimly lit, sound-attenuating room with response buttons under their hands. Visual stimuli were presented at the centre of the screen with a gray background. No seizures occurred in any of the patients during the preceding 12 h.

**Figure 1:**
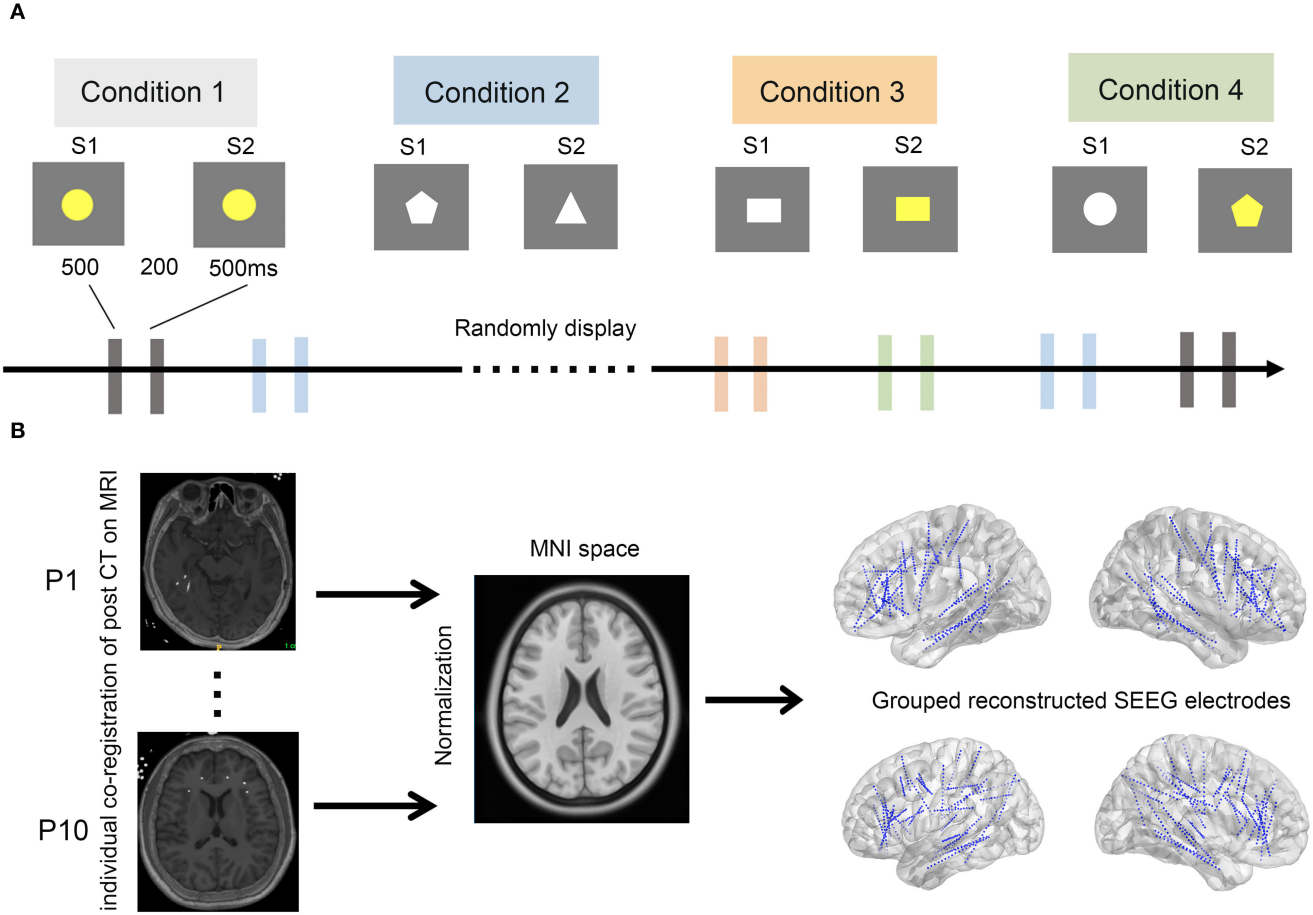
The experimental paradigm and pipeline of SEEG electrode reconstruction. **(A)** The experimental paradigm. Four conditions, including conditions 1–4, were randomly displayed every 5 s. A stimulus pair of stimulation 1 (S1) and 2 (S2) was sequentially presented in each condition. Of note, S1 and S2 were assigned as congruent, irrelevant incongruent, relevant incongruent, and conjunction incongruent in conditions 1–4, respectively. **(B)** Pipeline of SEEG electrode reconstruction. All implanted electrodes in all subjects were overlaid on a standardized brain in MNI space. Post-implantation CT images were individually co-registered to the pre-implantation MRIs (left), which were further normalized to standard MNI space (middle). The coordinates of each electrode were therefore calculated in the MNI space. Finally, all SEEG electrodes of the 10 patients were reconstructed and displayed over the three-dimensional brain in the MNI space (right).

A delayed match-to-sample paradigm was used in this study, which has been validated in our previous study and by other research groups. The stimuli consisted of different shapes with different colors. The visual stimuli presented to each patient were controlled by a stimulus system (STIM2; Neurosoft Labs Inc., Sterling, VA, USA). Each trial consisted of a pair of sequentially presented stimuli: a first stimulus (S1), followed by a second stimulus (S2). S1 and S2 were presented on the screen for 500 ms each, with an interval of 200 ms. The interval between each pair of stimuli was 5 s. The stimulus pairs were classified into four conditions of equal presenting probability: (i) S1 and S2 were identical (congruent condition); (ii) S2 was different from S1 in shape, same as S1 in color (irrelevant incongruent condition); (iii) S2 was different from S1 in color, same as S1 in shape (relevant incongruent condition); and (iv) different in both color and shape (conjunction incongruent condition) (Figure 1). The stimulus pairs of the four conditions were randomly presented in sequence. The whole task was divided into four blocks with a break of approximately 30 min in between to prevent fatigue in subjects. Patients were required to discriminate whether S1 and S2 were the same or not in color while ignoring their shapes and were instructed to press the corresponding keys on the keyboard as quickly and accurately as possible.

Stereoelectroencephalography data were recorded using the Neuroscan system (Scan 4.5; Neurosoft Labs Inc.) with a 128-channel SynAmps EEG/EP amplifier (Compumedics USA Inc., Charlotte, NC, USA) with a sampling rate of 2,000 Hz.

### Data Analysis of Local Field Potentials and Statistics

All SEEG data were processed using EEGLAB (https://sccn.ucsd.edu/eeglab/) and custom MATLAB codes unless stated otherwise.

Initially, the data were reviewed by visual inspection. The epoch that was contaminated with significant artifacts was discarded. Trials with incorrect responses and interictal epileptiform discharges were also excluded from further analysis. SEEG data were filtered with a bandpass of 0.5–150 Hz using a zero-phase shift finite impulse filter (Butterworth). After the notch filter at 50 Hz, epochs of all trials were extracted from 200 ms pre-S1 to 300 ms post-S2. Baseline correction was carried out using an interval of 200 ms (−200 ms pre-S1 to S1). The trials were automatically rejected when they contained neural activity that exceeded five standard deviations from the mean. More than 25 trials were averaged for each condition in each patient.

We next tested the significant differences between conditions. To address the spatial distribution of relevant incongruent processing, the responses on all the contacts were further classified according to the intrinsic functional networks. Using a two-sample *t*-test, two conditions, namely, congruent vs. irrelevant incongruent, congruent vs. relevant incongruent, and congruent vs. conjunction incongruent conditions of each contact within gray matter, were compared trail-by-trail in a time window of 0–800 ms from the point of S2 onset across each time point. Notably, the one-sample Kolmogorov–Smirnov test was performed to confirm the distribution of the experimental data. To refine the relevant incongruent effect, correlations of congruent vs. irrelevant incongruent and congruent vs. conjunction incongruent conditions were evaluated, ranging from 200 to 300 ms after S2, respectively. A value of *p* < 0.05 was considered to be statistically significant. To highlight spatiotemporal dynamics, a T map of relevant incongruent vs. congruent conditions was displayed (200–300 ms after S2 presentation).

Event-related potentials of each condition at all recorded sites were averaged and time-locked to S2 presentation. All data are presented as the mean ± SEM.

## Results

### Subject Characteristics and Behavior

In total, 10 patients (3 men and 7 women) aged 18–28 years (with an average age of 21.3 years) were included in our study (Table 1). In total, we obtained SEEG recordings from 888 recording sites on 62 electrodes (Figure 1).

**Table 1:** Basic information of the patients and the seizure onset zone.

**Patient number**	**Sex/Age**	**Hand laterality**	**Side of SEEG**	**Seizure onset zone**
1	F/24	Right	Bilateral	Left superior frontal gyrus
2	M/23	Right	Left	Left temporal lobe
3	F/18	Right	Bilateral	Right temporal and
				hippocampus
4	M/21	Right	Bilateral	Right frontal lobe
5	F/18	Right	Right	Right frontal lobe
6	F/19	Right	Left	Left temporal lobe
7	F/28	Right	Left	Left temporal and hippocampus
8	M/18	Right	Right	Right inferior frontal gyrus
9	F/21	Right	Right	Right temporal lobe
10	F/23	Right	Left	Left frontal pole

**Figure 2:**
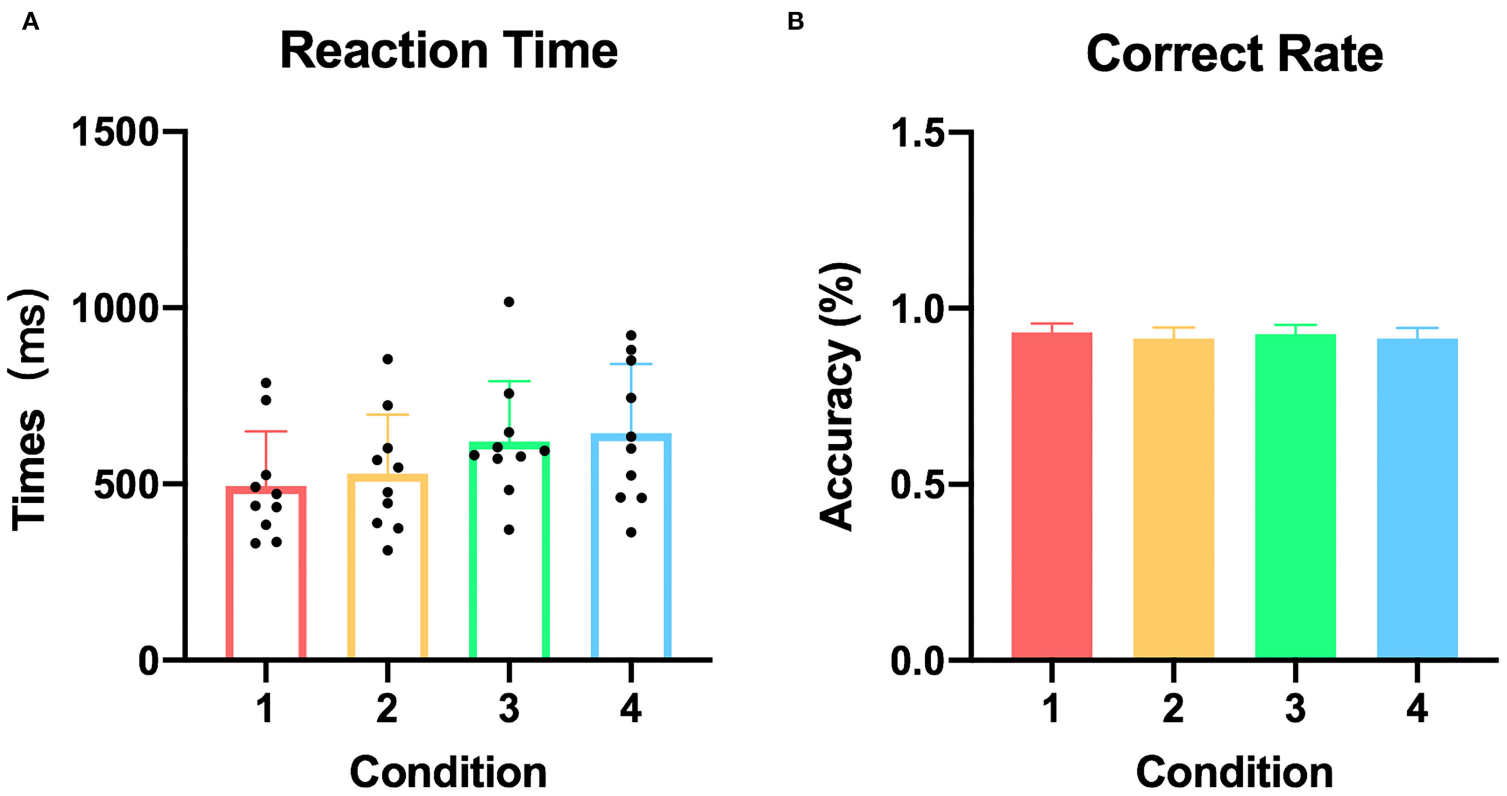
Reaction time and correct response rate of each condition. **(A)** The reaction time in all participants (condition 1: congruent, condition 2: irrelevant incongruent, condition 3: relevant incongruent, and condition 4: conjunction incongruent). **(B)** The correct response rate in all participants.

All participants performed the tasks successfully according to their behavioral performance with an average accuracy of 92.2%, and no difference was found among the four conditions (*p* = 0.952). In addition, the mean reaction times did not differ between the congruent condition with the other three incongruent conditions, respectively (p_condition1&condition2_ = 0.579, p_condition1&condition3_ = 0.063, p_condition1&condition4_ = 0.074) (Figure 2).

### Time Courses of Task-Evoked Perturbation Distributed in Different Brain Intrinsic Networks

**Figure 3:**
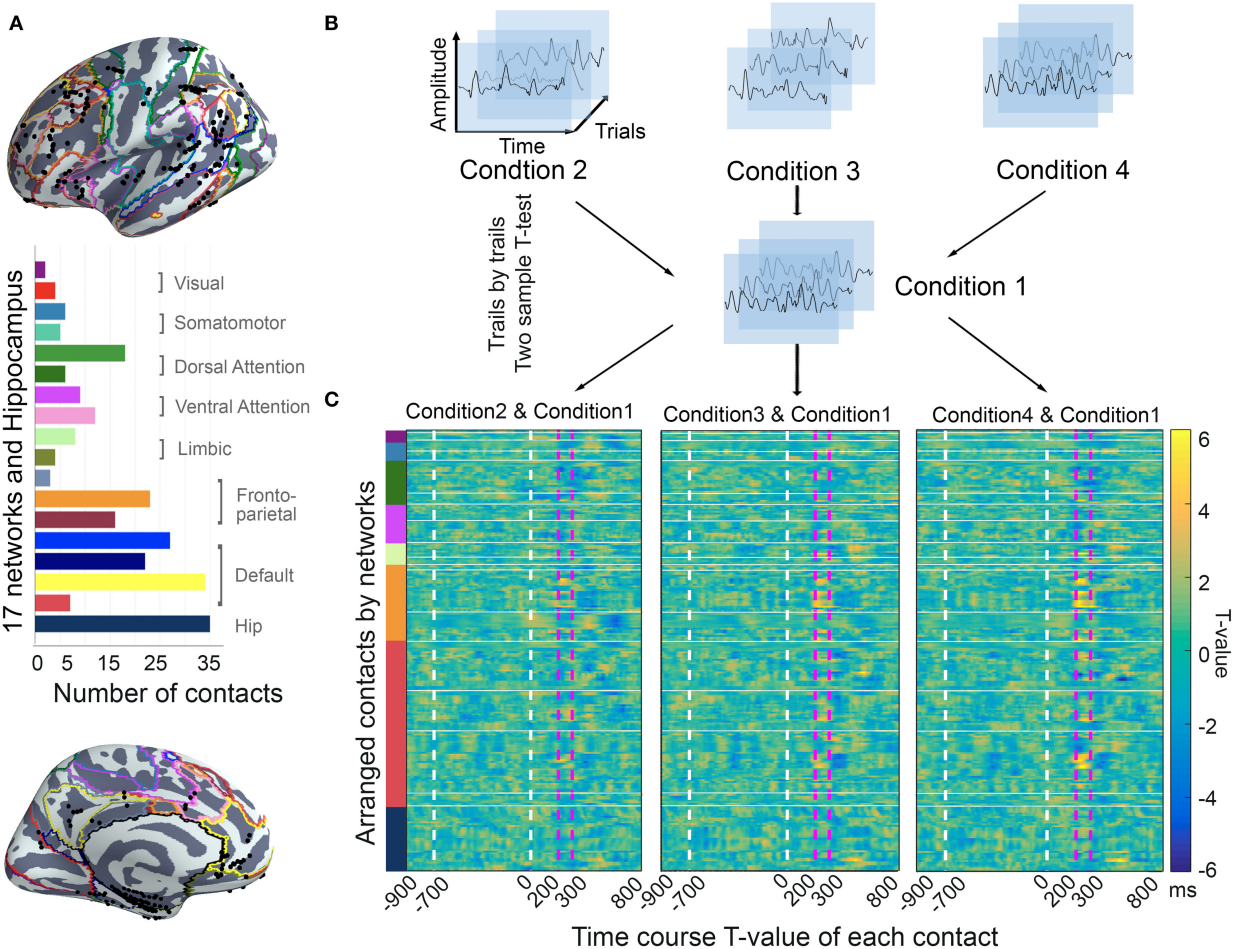
The location of contacts and time course *T*-value of each contact. **(A)** Location of contacts. Contacts (10 patients; *N* = 241) were located on the brain surface with 17 functional network parcellations in standardized MNI space. All the contacts were arrayed in 17-network and hippocampus. The bar plot shows the number of contacts within each brain region. **(B)** The ERP processing procedure. All the trials were assigned with stimulation time in each condition. The two-sample *t*-test was used to compare the time course task-evoked changes trail-by-trail. **(C)** The statistical test results for all contacts, which were arranged in the order of functional networks. The dashed white vertical lines in the plot show the S1 and S2 onsets, and the dashed pink vertical lines mark the time window of interest (200–300 ms after S2 onset).

According to the anatomical atlas of gray and white matter, 241 contacts within gray matter were included for subsequent analysis. Guided by intrinsic network anatomy from functional neuroimaging in healthy populations, the electrodes were distributed throughout multiple cortical locations and were divided into a 17-network and hippocampus subsystems (Figure 3).

Using the two-sample *t*-test, we screened all 241 contacts for the voltage of task-evoked changes in the intrinsic networks during the time window of −900 ms pre-S2 to 800 ms post-S2 presentation. Since our previous work showed that neural activity during 200–300 ms after S2 onset might be most relevant to incongruent information processing, thus we focused further analysis on this time window. Obviously, the *t*-values of the contacts varied over the distributed anatomical locations, and they showed distinct effects among the functional networks by visual inspection.

**Figure 4:**
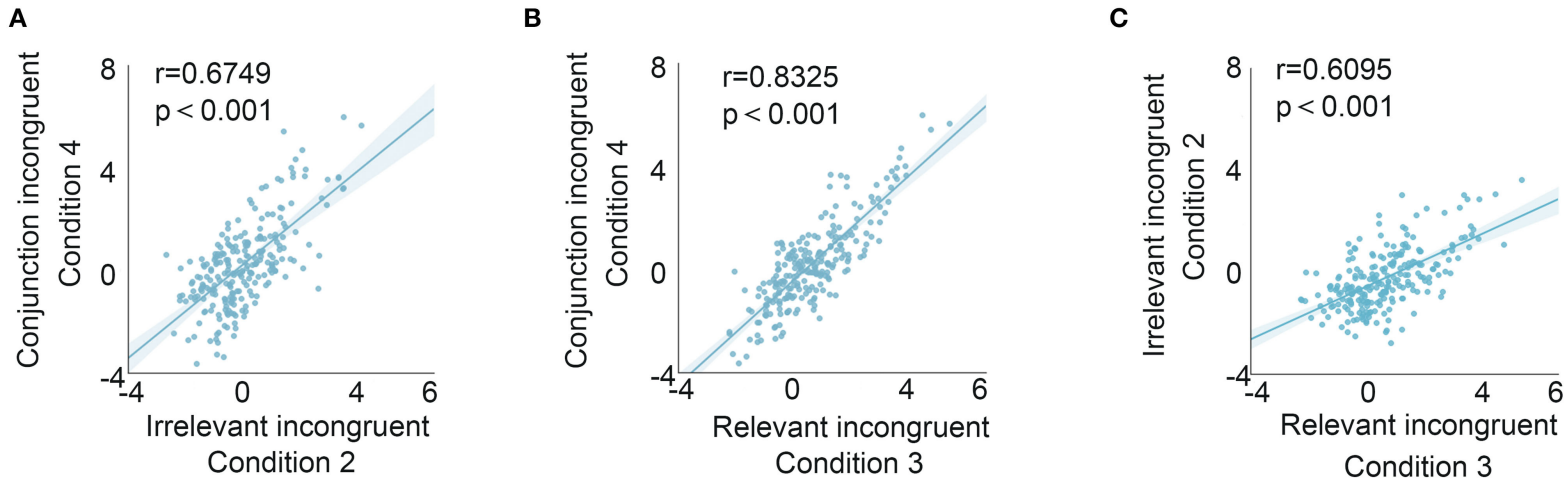
Correlation of different conditions (mean *t*-value from 200 to 300 ms post-S2 onset of each contact). The relationship between irrelevant incongruent and conjunction incongruent conditions **(A)**, the relationship between relevant incongruent and conjunction incongruent conditions **(B)**, and the relationship between relevant incongruent and irrelevant incongruent conditions **(C)**. The highest correlation was identified between relevant incongruent and conjunction incongruent conditions.

To better evaluate the relationship between the relevant incongruent condition and congruent condition, coefficients were attained by correlating the mean *t*-value (200–300 ms) between the relevant incongruent condition and irrelevant incongruent condition (0.6095, *p* < 0.001), the relevant incongruent condition and conjunction incongruent condition (0.8325, *p* < 0.001), and the irrelevant incongruent condition and conjunction incongruent condition (0.6749, *p* < 0.001), respectively, suggesting the distinct responses to the relevant incongruent information (Figure 4).

### Spatial Location of Different States in Incongruent Information Processing

#### Irrelevant Incongruent Information Processing With Automatic Detection

To clarify the different states of incongruent information processing, we found the anatomical location of each contact with significant statistics in those comparisons, which suggested that the brain areas are involved in the different states in incongruent information processing (Table 2).

**Table 2:** The anatomical location of different brain areas involved in the different states of incongruent information processing.

**Irrelevant incongruent & congruent**	**Relevant incongruent & congruent**
Superior parietal gyrus
Supramarginal gyrus	Supramarginal gyrus
Angular gyrus	Angular gyrus
	Middle frontal gyrus
	Superior temporal gyrus
	Middle temporal gyrus
Inferior temporal gyrus
Fusiform gyrus	Fusiform gyrus
	Posterior superior temporal sulcus
	Posterior cingulate cortex

First, when detecting task-irrelevant incongruence, the subjects responded after S2 appeared even if they did not notice the relevant incongruence, speculating that this process may belong to automatic detection. We calculated the mean *t*-value after S2 onset between the irrelevant incongruent condition and the congruent condition. The core brain areas were concentrated in the superior parietal lobule, inferior parietal lobule (supramarginal gyrus, angular gyrus), inferior temporal gyrus, and fusiform gyrus.

**Figure 5:**
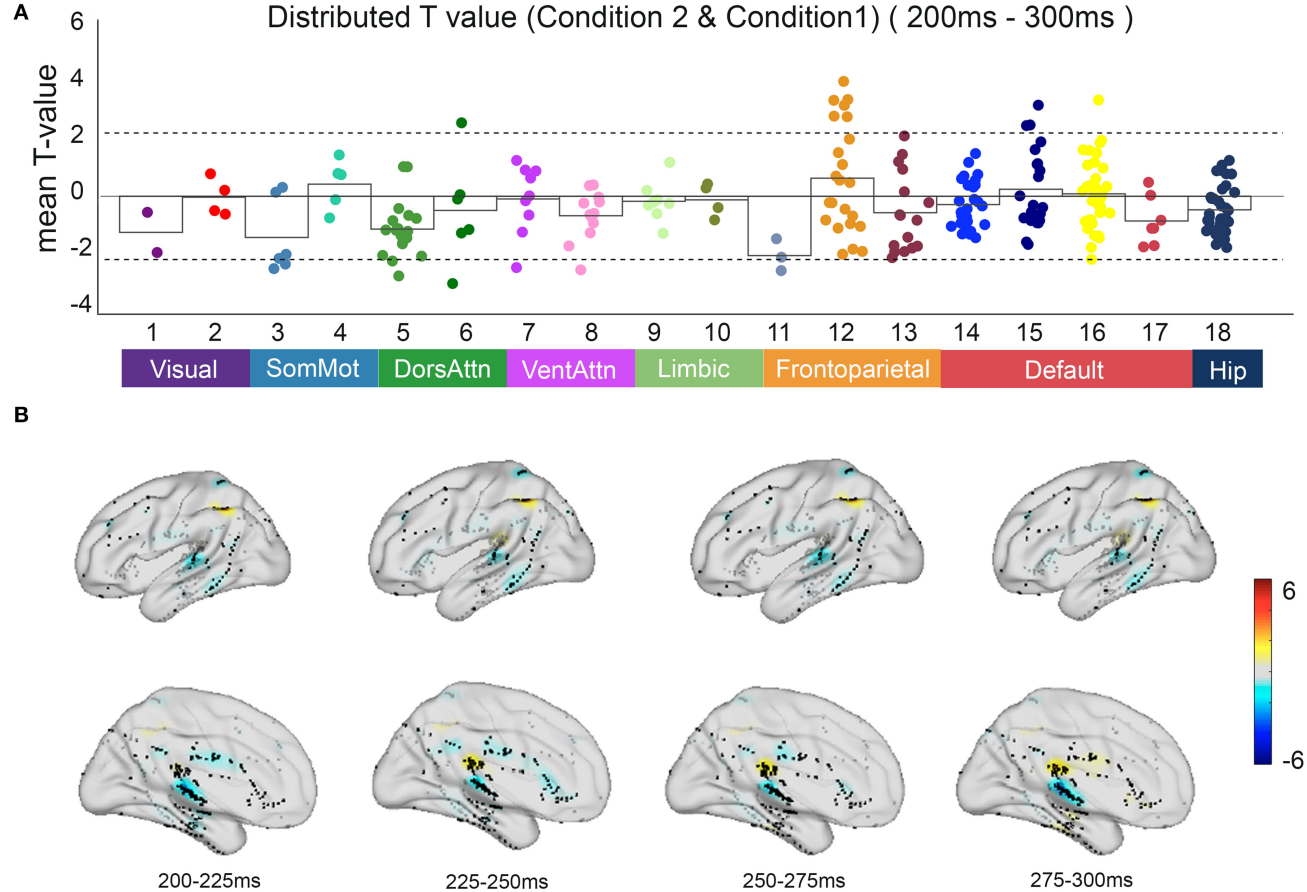
The neural response in functional networks and the spatiotemporal map of irrelevant incongruent condition. **(A)** Mean *t*-value distributed across 17 networks and hippocampus during 200–300 ms between the irrelevant incongruent condition and congruent condition. Significant responses were detected on the frontoparietal control network and default mode network (*t*-value > 2, *p* < 0.05). **(B)** The *t*-value map shows dynamics between the irrelevant incongruent condition and congruent condition. Color intensity indicates *t*-value of positive (red) or negative (blue).

Based on the anatomical boundaries defined by the literature, contacts were categorized into the 17-network and hippocampus subsystems. Focusing on the irrelevant incongruent condition, the mean *t*-value compared with the congruent condition between 200 and 300 ms after S2 onset in all contacts indicated the specificity of spatial distribution in the parietal regions of the frontoparietal control network (FPCN) and temporal regions of the default mode network (DMN) (Figure 5).

#### Relevant Incongruent Information Processing With Controlled Discrimination

Furthermore, in the relevant incongruent condition, the subjects needed to identify whether the color feature was different between S1 and S2 in the relevant incongruent condition and the conjunction incongruent condition. The relevant incongruent information processing was referred to as controlled discrimination. We identified that the dynamics of task-evoked perturbation over a long period of time were more specific to incongruent information processing than to congruent information processing.

**Figure 6:**
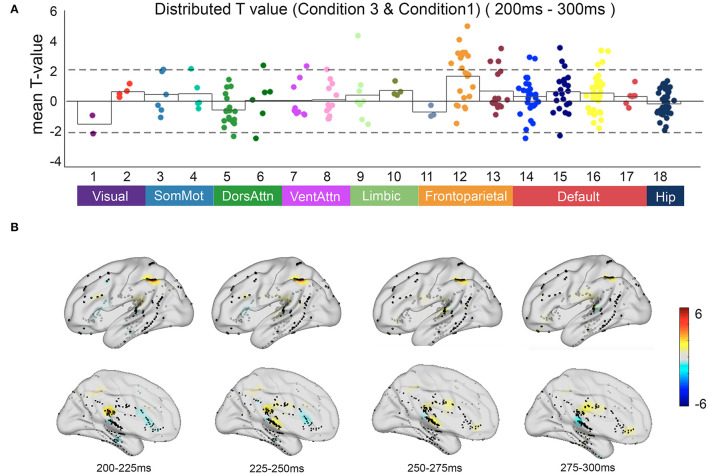
The neural response in functional networks and the spatiotemporal map of relevant incongruent condition. **(A)** Mean *t*-value distributed across 17 networks and hippocampus during 200–300 ms between the relevant incongruent condition and congruent condition. Significant responses were detected on frontoparietal control network and default mode network (*t*-value > 2, *p* < 0.05). **(B)** The *t*-value map shows dynamics between the relevant incongruent condition and congruent condition. Color intensity indicates *t*-value of positive (red) or negative (blue).

Compared with automatic detection, the brain regions that were activated for controlled discrimination included the frontal middle gyrus, superior temporal gyrus, middle temporal gyrus, posterior superior temporal sulcus, and posterior cingulate cortex. The T map showed the frontal regions of the FPCN and the PCC within the DMN engaged in the relevant incongruent information processing with statistical significance (*p* < 0.05) (Figure 6).

**Figure 7:**
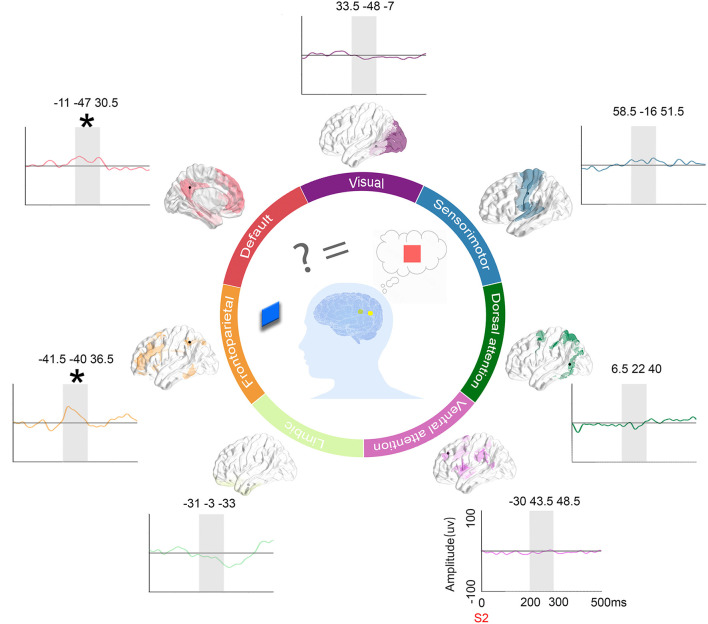
ERPs on representative contacts over different functional networks and schematics. Location of contacts: the black dot represents the contacts on the parcellation maps of the 7 functional networks. Subtract ERPs morphology: the mean ERP amplitude of the congruent condition subtracted from the other incongruent conditions (orange: the congruent condition vs. irrelevant incongruent condition, green: the congruent condition vs. relevant incongruent condition, and purple: the congruent condition vs. conjunction incongruent condition) in the time window of interest (200–300 ms in gray shadow) in 7 networks. Schematic figures: The schematic picture shows the activation when people need to integrate incoming extrinsic information (red square) with prior intrinsic information (blue square).

To highlight the specific neural responses to relevant incongruent information, representative ERPs (condition 3 of relevant incongruent task minus condition 1 of congruent task) among different intrinsic functional networks were picked up for visual inspection. The results are displayed in Figure 7. Finally, schematic figures were conceived to characterize the dynamic profile of the human brain.

## Discussion

Based on the direct recording of neural activity, our study reveals several vital points in incongruent information processing. First, we demonstrated the cortical dynamics with high temporal and spatial resolutions during the delayed match-to-sample task. Our data indicated that the N2 subcomponents are responsible for the incongruent stimulus, consistent with previous findings (Bennett et al., 2014). Second, we identified the critical areas and networks involved in the processing of irrelevant and relevant incongruent information. Moreover, we suggested that there may be two integrated modalities of incongruent information processing in different states referred to as automatic detection and controlled discrimination.

A negative peak between 200 and 350 ms in scalp recording is defined as N2 since it follows a negative peak at ~100 ms in the auditory modality or at around approximately 180 ms in the visual modality (Donkers and van Boxtel, 2004; Sur and Sinha, 2009). Hence, there has been an increased interest in the N2 components for understanding the nature and sequence of cognitive control that covers strategic monitoring and control of motor responses in recent years (Folstein and Van Petten, 2008). In fact, N2 comprises several subcomponents (N2a, N2b, and N2c) that can be elicited in different tasks under various conditions. More studies have focused on the N2a, which was also termed auditory mismatch negativity (MMN) (Naatanen et al., 2007; Fishman, 2014), the N2b, which was consistently observed in oddball studies in the visual modality (Szucs et al., 2007), and the N2c, which was distinguished from the MMN and N2b and was modality-specific on scalp distribution, posteriorly in the visual modality but at the frontocentral area in the auditory modality. The scalp EEG results showed a large component that was sensitive to incongruent information and could be elicited during the interval of 200–300 ms after S2 presentation, i.e., in a pair of sequentially presented stimuli, the second stimulus was different from the first stimulus no matter what the attributes of the stimuli are, such as shapes, colors, numbers, and locations. Typically, participants judged whether two consecutively presented stimuli were congruent or incongruent in specific attributes in the delayed match-to-sample task (Szucs et al., 2007).

Such an incongruent-related N2 component was thought to reflect a process of stimulus discrimination, whereas the MMN complex and N2b complex were thought to index different stages of mismatch detection (Wu et al., 2010). In particular, N270, a negative ERP component with a peak latency of ~270 ms, is speculated to reflect the processing of incongruent information and be influenced by attention. As a result, we indicated that the N2 subcomponents are responsible for the incongruent stimulus in the sequential matching paradigm, which was consistently confirmed by a series of studies (Wang et al., 1998, 2000, 2003; Cui et al., 2000; Bennett et al., 2014; Liang et al., 2017).

In this study, the application of SEEG offered the opportunity to probe the processing with high temporal and spatial resolutions. Technically, the bipolar montage of consecutive adjacent contacts is traditionally employed to attenuate the volume effects. Nevertheless, the polar of N2 recorded intracranially was different from that of scalp EEG. Hence, we compared the evoked potentials with baseline activity and tested for significance by utilizing a two-sample *t*-test for every data point between the two conditions, which was data-driven and did not make any assumptions about when the effect was expected (Guthrie and Buchwald, 1991; Piai et al., 2015). The significance between conditions was speculated to avoid the polar issue.

Our data showed the specific responses to both irrelevant and relevant incongruent tasks. Prominent statistical significance was detected in the incongruent task specifically compared with the congruent task between 200 and 300 ms after the S2 presentation. Importantly, our research suggests that different brain regions are responsible for different states of incongruent information.

In the irrelevant incongruent condition, although we ignored the feature of shape, there were also significant changes when compared with the congruent condition. Our results demonstrated that some essential areas, the superior parietal lobule, inferior parietal lobule (supramarginal gyrus and angular gyrus), inferior temporal gyrus, and fusiform gyrus, were significantly activated during this processing. Studies from primates have shown that parietal neurons are activated earlier during bottom-up attention (Buschman and Miller, 2007). Evidence from neuroimaging studies suggests that the inferior parietal lobule participates in the bottom-up perception that is uniquely human (Igelstrom and Graziano, 2017). Notably, a similar SEEG study revealed the contribution of the rostral inferior parietal lobule in decision processing (Ter Wal et al., 2020). Particularly, more recent studies revealed that human parietal areas played a central role in feature detection decisions (Guidotti et al., 2019), which is assumed to be integrated into incongruent condition control processing. The inferior temporal gyrus, as part of the visual ventral pathway, is associated with object, face, and scene perception, and studies of single-cell electrophysiological recordings from primates directly confirm the involvement of the inferior temporal gyrus in object recognition (Gross, 2008; Conway, 2018). From the view of functional networks, the inferior parietal lobule is a part of the FPCN, which is considered to support cognitive control and decision-making processes (Martin-Signes et al., 2019). Meanwhile, the temporal regions of the DMN were also involved in the processing of irrelevant incongruent information, which indicated that the detection of irrelevant incongruent information may be automatic.

Moreover, by comparing the relevant incongruent condition with the congruent condition, we speculated that the activated brain regions were involved in the feature-controlled discrimination processing, including the inferior parietal lobule (supramarginal gyrus and angular gyrus), middle frontal gyrus, superior temporal gyrus, middle temporal gyrus, fusiform gyrus, posterior superior temporal sulcus, and posterior cingulate cortex. The frontal regions, such as the middle frontal gyrus, are part of the FPCN, which plays a key role in the top-down control of attention. Additionally, the middle frontal gyrus is considered to be the convergence point of the dorsal and ventral attention networks. In addition, the superior temporal gyrus, middle temporal gyrus, fusiform gyrus, and posterior superior temporal sulcus participated in the relevant incongruence feature discrimination. Interestingly, we detected the involvement of the PCC in goal-driven incongruent information attention, which has not yet been described. The PCC is one of the cores of the DMN, which may be related to the problem-solving and executive control processes (Andrews-Hanna et al., 2010; Leech and Sharp, 2014). In addition to the classical concept that DMN increases its activity during passive states and decreases its activity during positive states, recent studies reported the DMN as an active and dynamic “sense-making” network that integrates incoming extrinsic information with prior intrinsic information (Yeshurun et al., 2021). In our paradigm, the S1 stimulus represents the intrinsic information, and the S2 stimulus represents the incoming information. The results suggested that different brain regions are significantly activated when processing target-relevant incongruent information, which indicated that the discrimination processing of relevant incongruent information may require controlled attention.

There are also some limitations in our research. First, the potential impact of an altered brain network should be noted because the participants were patients with presurgical epilepsy. To minimize the confounding effects of epilepsy on the acquired intracranial electrophysiological data, rigorous methods were adopted in this study: only patients with focal epilepsies who have normal intelligence were studied; only patients with a few localized deficits were included; all obtained data were several hours outside the window of seizures; and only the channels that are free of pathological activity were included at the individualized level (Parvizi and Kastner, 2018). Second, the number of patients was limited. Accordingly, the brain regions were not covered completely. For example, SEEG electrodes were sparsely implanted in the dorsal lateral prefrontal cortex and dorsal anterior cingulate in our patient group since the placement of the electrodes was determined by clinical ground only.

In summary, this study showed the fine-grained dynamics of incongruent information processing, and our results offer new insights into the refined understanding of how the brain processes incongruent information. We suggested that there may be two integrated modalities of incongruent information processing in different states referred to as automatic detection and controlled discrimination. The process of automatic detection of disparity information requires the involvement of a few brain regions with networks, such as the parietal regions of the FPCN and inferior temporal gyrus of the DMN. Furthermore, controlled discrimination of incongruence information requires the involvement of wider networks and a wide range of nodes in the network, including the frontal lobe of the FPCN and the PCC of the DMN. There may be a spatiotemporal interaction between the networks in incongruent information processing. The integrated brain network collaboration model may be an important modal of the cognitive control process that deserves more research in the future.

## Data Availability Statement

The raw data supporting the conclusions of this article will be made available by the authors, without undue reservation.

## Ethics Statement

The studies involving human participants were reviewed and approved by Xuanwu Hospital, Capital Medical University. The patients/participants provided their written informed consent to participate in this study.

## Significance Statement

It is the fundamental ability to discriminate incongruent information through the attentional biasing of perceptual processing. Scalp electroencephalography and neuroimaging offer an optimized approach to probe the time course of incongruent information processing. However, the knowledge of such neural dynamics has not yet been defined. Therefore, it is necessary to refine how the brain processes incongruent information. We used SEEG recordings from patients with intractable focal epilepsy during the delayed match-to-sample task and demonstrated the cortical dynamics with high temporal and spatial resolutions. Moreover, we identified the critical cortical areas processing the incongruent information over the frontoparietal control network and default mode network. Our results will offer new insights into the refined understanding of how the brain processes incongruent information.

## Author Contributions

JD: formal analysis and writing—original draft. YZ and DY: investigation and editing. LR: methodology and writing—review and editing. TY and XZ: resources. LR and YW: conceptualization and supervision. All authors contributed to the article and approved the submitted version.

## Funding

This work was supported by grants from the National Natural Science Foundation of China (Nos. 81771398 and 82071454) and the Beijing Natural Science Foundation (No. 7202062). This work is also supported by National Science and Technology Resources Sharing Service Platform Program (2016YFC1000307).

## Conflict of Interest

The authors declare that the research was conducted in the absence of any commercial or financial relationships that could be construed as a potential conflict of interest.

## Publisher's Note

All claims expressed in this article are solely those of the authors and do not necessarily represent those of their affiliated organizations, or those of the publisher, the editors and the reviewers. Any product that may be evaluated in this article, or claim that may be made by its manufacturer, is not guaranteed or endorsed by the publisher.
